# Increasing cultural awareness: qualitative study of nurses’ perceptions about cultural competence training

**DOI:** 10.1186/s12912-019-0363-x

**Published:** 2019-08-22

**Authors:** Anu-Marja Kaihlanen, Laura Hietapakka, Tarja Heponiemi

**Affiliations:** National Institute of Health and Welfare, P.O. Box 30, FI-00271 Helsinki, Finland

**Keywords:** Healthcare professionals, Nurse, Cultural awareness, Cultural competence, Training

## Abstract

**Background:**

Nowadays, healthcare professionals worldwide deliver care for increasing numbers of culturally and linguistically diverse patients. The importance of cultural competence is evident in terms of the quality of healthcare, and more knowledge is needed about different educational models and approaches that aim to increase cultural competence. This study examines the perceptions of nurses about the content and utility of cultural competence training that focuses on increasing awareness of one’s own cultural features.

**Methods:**

The training was conducted at one primary care hospital in southern Finland. Participants were registered nurses (*n* = 14) and practical nurses (*n* = 6) from different hospital units. Four 4-h training sessions—including lectures, discussions and short web-based learning tasks—were arranged during a four-week period. Semi-structured, small group interviews were conducted with 10 participants to examine their perceptions about the content and utility of the training. Qualitative content analysis with a conventional approach was used to analyse the data.

**Results:**

Perceptions about the training were divided into three main categories: general utility of the training, personal utility of the training, and utility of the training for patients. General utility pertains to the general approach that the training provided on cross-cultural care, the possibility to initiate an open discussion, and the opportunity to improve current practices. Personal utility pertains to the opportunity to become aware of one’s own cultural features, to change one’s way of thinking, to obtain a new perspective on one’s own communication practices and to receive justification for carrying out particular workable practices. Utility for patients pertains to fostering better awareness and acknowledgement of patients’ differing cultural features and an increased respect in healthcare delivery. Additionally, the quality of the training was highlighted, and suggestions for improvement were offered.

**Conclusion:**

Training that increases healthcare professionals’ awareness of their own cultural features was perceived as useful and thought-provoking. Increased awareness might facilitate the communication between healthcare professionals and patients, which is a crucial component of quality healthcare. It seems that in the future, training opportunities that allow larger groups to participate are needed, regardless of the time and place, and utilising the potential of e-learning should be considered.

## Background

Healthcare professionals worldwide are required to deliver care for an increasing number of culturally and linguistically diverse patients. Problems related to language and cultural issues are recognised as a threat to patients’ safety in hospitals [1] and the concept of cultural competence has gained attention as a strategy to provide equal and quality healthcare services for culturally diverse patient groups [2]. Cultural competence is known as a multi-dimensional construct, but it typically refers to a person’s cultural sensitivity or attitudes, cultural awareness and cultural knowledge and skills [3–5]. In the healthcare setting, cultural competence is defined as an understanding of how social and cultural factors influence the health beliefs and behaviours of patients and how these factors are considered at different levels of a healthcare delivery system to assure quality healthcare [6].

Effective communication between healthcare providers and patients is known to be necessary for quality healthcare [7]. A large number of culturally diverse patients often present communication challenges for healthcare delivery, especially if sociocultural differences are not completely accepted, appreciated, explored or understood [6]. A lack of cultural understanding increases negative attitudes towards cross-cultural care and also affects healthcare professionals’ perceived preparedness to take care of culturally diverse patients [8]. Moreover, anxiety about interacting with people from different cultures has an influence on a person’s level of engagement in intercultural communication [9]. And when combined with uncertainty, it further decreases effective communication and can lead to the increased use of stereotypes [10]. In contrast, an increased awareness about the sociocultural components of illness as well as reflecting on a healthcare professional’s own strengths and weaknesses when communicating with different populations are seen as key to overcoming different communication difficulties [11].

During the past decade, the need to increase the cultural competence of healthcare staff has been clearly recognised. This can be seen in the number of educational interventions and training programs that have been developed to improve the knowledge and skills essential to understanding and managing sociocultural issues in a healthcare setting [6, 12]. To be able to improve and sustain the cultural competency of healthcare professionals, training should be offered throughout a professional’s career [2, 12], tailored to take into account individual and organisational contexts [13] and involving key stakeholders in the design, implementation and evaluation of the programs [14, 15]. It is further recommended that both standard cultural competence training as well as more situation-specific training should be provided [2].

Even though there is currently little evidence about the effectiveness of cultural competence training on patient-related outcomes [14, 16], there is clear evidence about the positive effects of these interventions on healthcare professionals’ attitudes, knowledge and behaviour with respect to cross-cultural care [5, 13]. However, more knowledge is still needed to determine which educational models are most effective and feasible in what specific contexts and groups and how many resources (e.g. time) should be allotted for reaching the desired outcomes [13]. This qualitative study was conducted to examine the perceptions of nurses regarding the content, utility and implementation of cultural competence training that aimed to ease cross-cultural encounters by increasing awareness of one’s own cultural features. The goal was to gain knowledge that can be used in the development of national cultural competence training to healthcare professionals.

## Methods

### Setting and participants

The study was conducted in one large primary care hospital in southern Finland in autumn 2017. This hospital was chosen because it is located in an area that has a large number of immigrants (1/4 of all immigrants living in Finland). In 2017, 16% of the population in this area were foreign-language speakers (compared with 7% in the total population of Finland). The largest groups were Russian, Estonian and Arabic speakers [17]. An invitation to participate in the training was delivered to healthcare professionals in the hospital by the ward managers. Participants were expected to be physicians, registered nurses or licensed practical nurses with prior experience in taking care of culturally diverse patients. A group of 20 registered nurses (*n* = 14) and practical nurses (*n* = 6) from seven different units were enrolled in the training. At the end of the training, an email was sent to all participants with an invitation to participate in small group interviews. Ten (*n* = 10) participants responded and were willing to participate.

### Cultural competence training

Cultural awareness was chosen as the main construct for the training because self-reflection on one’s own culture can be seen as an important component of cultural competence, and understanding one’s own cultural features and values helps in understanding the beliefs, values and behaviour of others [18]. Cultural awareness is one component of Campinha-Bacote’s (2002) model of cultural competence in healthcare delivery, which explains cultural competence as a process that requires healthcare workers to engage in an active and ongoing effort to achieve the ability to provide culturally responsive healthcare services [18]. Instead of providing culturally specific facts about other cultures—which can increase the use of stereotypes [5]—the training was designed to take a more general approach to cultures, with the main goal being to increase awareness of different cultures by scrutinizing one’s own cultural features. In order to develop training that takes into account the context and involvement of key stakeholders, we utilised a wide range of sources in the development. The content of the training was based on (a) the theoretical literature about the different cultural dimensions (e.g. differences in cultural values, such as individualism vs. collectivism, power distance or orientation in time) [19, 20]; (b) several research articles regarding cultural pain, differences in personal space, and the importance of considering the spiritual needs of foreign patients [21–23]; (c) knowledge obtained from different cultural experts such as a priest and personnel from the Centre for Torture Survivors in Finland; and (d) knowledge obtained from our previous interview study. Interviews with 25 Finnish healthcare professionals were conducted in order to examine the main challenges that such healthcare professionals (nurses, doctors and dentists) face when taking care of culturally diverse patients [24]. Additionally, these interviews assessed perceived educational needs. The interviews revealed that the challenges are mainly related to communication between the patients and healthcare professionals, including language barriers, problems with visitors, gender issues and differences in pain interpretation. Perceived educational needs related to gaining an understanding of patients experiences with the Finnish healthcare system, the need to share experiences with colleagues about cross-cultural care, and learning some culture-specific facts or guidelines that could help in everyday nursing practice.

Constructivism learning theory was chosen as the pedagogical approach because it highlights the activity and engagement of the learner in using one’s own prior experiences in constructing new knowledge, developing an understanding, and making meanings [25]. The participants were encouraged to reflect about their prior experiences and encounters with culturally diverse patients and discuss in groups in order to inspire further thinking. The training included 16 h of face-to-face teaching, which was divided into four 4-h sessions and arranged for 4 weeks. The sessions were arranged once a week to give participants an opportunity to ponder and assimilate the learned content in their daily work before the next session. Participants attended the sessions during their working hours, so afternoon times were chosen. It was believed that afternoon times would improve participants’ opportunities to attend the sessions because more staff was present in the wards then.

The sessions were designed to move from the theoretical level to the practical level, and each session built upon the previous one. The main teaching method was adapted from ‘storytelling’, wherein the educator—an experienced teacher from a multicultural centre—used real-life examples, stories and pictures to demonstrate different cultural aspects. Storytelling was used because of its strength in promoting the adoption of multiple viewpoints and making sense of unknown theoretical situations, norms and values by using real-life experiences [26]. For example, the teacher described situations where differences in the way of communication (regardless of the language) have created unexpected misunderstandings. Furthermore, the teacher showed pictures that demonstrated how differently people with different cultural backgrounds can perceive the same images. Each session also included group discussions and learning tasks such as construing personal factors behind one’s own cultural features in order to become aware of the cultural diversity and to understand why culture-specific ‘facts’ cannot be used in patient care. Web-based learning platforms such as Padlet (an on-line post-it board) were also utilised, as they allowed the participants to share their thoughts anonymously with others. A description of the contents of the sessions is presented in Table 1.

**Table 1:** Contents of the sessions

Session	Content of the session
1. “What is Culture?”	-Different cultural dimensions and how these dimensions occur in our everyday life and in healthcare.
2. “Culture in me”	-Significance of being aware of one’s own cultural features in order to be able to understand others. How are our own cultural features constructed, and how are they seen in healthcare work? -Why are cultural ‘facts’ or assumptions not applicable in patient care? -Cultural pain. How do background and previous experiences affect pain interpretation? -Cultural ‘cage’. How does it regulate our behaviour towards others?
3. “Communication”	-Personal space. How can it be communicated to others? -What are our own communication features and challenges? -How do cultural values affect our way of communicating? -What is good and understandable communication with patients from different cultural backgrounds? -What issues typically mess up or complicate the communication process?
4. “Meaning of conviction”	-What is our own attitude towards spiritualism? What can different attitudes mean in a healthcare context? -Interaction between culture and religion. Does culture generate religion, or is it the other way around? -How can we value a patient’s convictions and spirituality? ➔Introduction to a conversational model (opening model) that can be used to assess patients’ spiritual needs

### Data collection

After the final training session, three semi-structured small group interviews (*n* = 4 + 2 + 3) and one single interview (*n* = 1) were conducted in the hospital to explore the perceptions of the participants about training. Five (*n* = 5) of the interviewees had attended all of the training sessions, three (*n* = 3) had attended three sessions, and two (*n* = 2) had attended two sessions.

Two researchers with a background in nursing and prior experience with interview studies conducted the interviews. The interviewers were familiar with the content of the training, as they had been present at each training session. The participants were asked questions such as how they perceived the content of the training, what they found useful or not useful in the training and why, whether something was missing from the training, and how they perceived the overall implementation of the training including the learning methods and the timing and length of the sessions. The interviews lasted 30–40 min and were audio-recorded and transcribed verbatim for the analysis. Field notes, such as demographics of the participants and the main points from each interview, were also taken during the interviews and used afterwards in the reflective discussion between the two interviewers [27].

### Data analysis

Qualitative content analysis with a conventional approach was used to analyse the data. The method is suitable for interview data collected from open-ended questions, and it allows the researcher(s) to explore personal perceptions without resorting to preconceived categories [28]. First, the interview transcripts were read through several times to obtain a picture of the data in its entirety. After familiarising ourselves with the data, the transcripts were read again to code all the expressions from the text that described participants’ perceptions of the training. The length of the codes (the units of analysis) varied between a few words and a few sentences. While coding, notes were also made about first thoughts and impressions. Next, codes with similar content were grouped as subcategories, which were given a descriptive name. Finally, subcategories that had the same perspective were then grouped into five main categories (Table 2). One researcher made the initial categorisation, which was then discussed and verified by another researcher (who was also present during the data collection phase, had the field notes from interviews, and was familiar with the data).

**Table 2:** Categorisation of the perceptions of the training

Main category	Subcategory
General utility	General perspective on cultural issues
	Starting an open discussion about cultural issues
	Opportunity to improve current practices
Personal utility	Opportunity to become aware of one’s own cultural features
	Change one’s way of thinking
	Obtaining a new perspective on one’s own communication practices
	Justification for carrying out workable practices
Utility for patients	Better awareness and acknowledgement of patients’ differing cultural features
	Increased respect in healthcare delivery
Quality of training	Serves the needs of learners
	Expertise of the training provider
	Excellent teaching skills of the educator
Suggestions for training improvement	Listening to persons from different immigrant groups
	Condensed or partly Web-based training to ease participation
	Written summary from each training session
	Rules and customs of different religions

## Results

The participants were registered nurses (*n* = 8) and licensed practical nurses (*n* = 2) from five different hospital wards. Most of the participants were female (*n* = 9), 23 to 55 years old (average age of 37). Their work experience in the healthcare field varied between 2 and 33 years (average 14 years). None of the participants had previously attended a cultural competence training designed to address cross-cultural care or multicultural issues. The participants reported whether they encounter patients from different cultural and linguistic backgrounds on a daily (*n* = 3), weekly (*n* = 4) or monthly (*n* = 3) basis.

We divided the participants’ perceptions of the training into three main categories: general utility, personal utility, and utility of the training for patients. The participants’ perceptions of how the training had been implemented were divided into two categories: quality of the training and suggestions for improvement. Each main category had two to four subcategories (Table 2).

### General utility

Participants expressed that they were pleased that the cultural competence training had provided them with a more general, rather than entirely a healthcare-orientated, perspective on cultural issues. The fact that the educator in charge was not a healthcare professional was seen as an advantage because she was able to bring new ideas and viewpoints into the hospital environment. Participants also stated that they were pleased that many of the real-life examples presented in the lectures were not from the healthcare environment but dealt with more general incidences from everyday life.

*‘Usually we are educated by nurses or some other healthcare professionals. They are so close to us, and the hospital environment, that they can be as blind as we might be in these matters.’ (i1, n4)*

The participants saw the training as an important opportunity to start a general and open discussion about cultural issues and, for example, about conviction, which workers typically avoid discussing and which is not part of the general work culture. Having the possibility to share their thoughts with colleagues was highly appreciated, and the small group and engaging lecturing style of the educator seemed to facilitate participants’ involvement in the discussions.

*‘The atmosphere was open and, because we were a small group, it was easy to interact. I realised that people rarely dare to speak up and discuss [things] as freely as we did. Usually people just sit quietly in these training [situations].’ (i2, n2)*

Participants described the training as an opportunity to develop their current healthcare practices. In order to achieve any general improvements, they thought that the whole healthcare organisation should have the opportunity to attend such trainings. Participants also noted their own responsibility in making improvements, and they stated they were enthusiastic to share the learned knowledge with their co-workers. However, such sharing was noted to be challenging because increasing cultural awareness was primarily seen as an individual process.

*‘It was difficult to tell others what was discussed in the lectures. The knowledge didn’t just come from the sentences that we heard. It was also behind the sentences and cannot be explained with words. When I tried to describe these things to others, the message [got] changed along the way.’ (i1, n1)*

### Personal utility

The training was described as an important opportunity to become aware of one’s own cultural features. The participants realized the extent to which their own cultural ‘cage’ guided their behaviour, and how it also affects the way they interpret the behaviour of others. Subsequently, the participants noted changes in their way of thinking. They felt more open-minded; and they reported that after the training, they had started paying more attention to the way they acted when taking care of culturally diverse patients. Participants felt that the training provided them many new, even surprising, perspectives about their own daily communication patterns. Realising the common features of their communication patterns, and how they might complicate their interactions with patients, allowed them to develop their communication skills.

*‘Training really helped me to understand that that’s exactly how we act, and maybe we should try to act a bit differently … pay more attention to how we talk and interact with others.’ (i2, n1)*

*‘I really wasn’t aware that we often communicate with silence, [our] eyes, etc. … and how much we tend to communicate between the lines. These things had never crossed my mind because they’re so automatic.’ (i1, n2)*

Despite the fact that several participants expressed a need to develop current practices and their own way of acting, many participants also perceived the training as a justification for carrying out certain practices that they feel are important with respect to established customs, regardless of the culture of the patient. The participants also reported that their courage to encounter culturally diverse patients increased as a result of the training.

*‘Sometimes I feel that female patients’ husbands or relatives speak for the patients. I think that every patient must have a right to speak up, and the training gave me courage to stick with this principle and say, “In here, we would like to hear [from] the patient alone, therefore, could you please give us a minute … ”’ (i3, n1)*

### Utility of the training for patients

The participants reported that the training had utility value for the patients as a result of nurses having a better awareness of and ability to acknowledge the differing cultural backgrounds of particular patients. For example, participants stated that they had started paying more attention to supporting the communality of certain patient groups after the training.

*‘Many cultures are so much more communal than we are. People also want to take care of their relatives when they are in the hospital, and I want to support that. We should try to learn from that.’ (i1, n4)*

Additionally, participants reported that the training had increased the respect that culturally diverse patients receive when seeking healthcare. The participants emphasised the importance of providing equal treatment and being respectful and non-judgmental of others, especially when the customs of certain cultures differ from one’s own ideology.

*‘Even if the patient and his or her relatives, family situations or way of living goes against my cultural beliefs, it doesn’t mean that I have a right to discriminate against them. For example, in some cultures, girls get married young and men have power in decision making. Despite (the fact that) that’s not happening in my life, in my country or in my culture, it doesn’t make it wrong, and I have to respect that. The training gave me the tools to think about these things.’ (i3, n1)*

### Quality of the training

The participants felt that the training was of a high quality, and many stated that the training had exceeded their expectations. They also noted the importance of providing training that serves the needs of the learners and that it is highly important to consider the starting level of the learner when designing the training. Participants were mostly satisfied with the contents of the sessions, but many felt the discussion model in the conviction session was unnecessary or too straightforward. Instead of using any pre-specified phrases, nurses felt that it is better to be sensitive to the situation and use their professional skills as nurses when discovering patient’s spiritual needs.

*‘I feel that as a nurse, and after the nursing education [that] I have completed, I must be able to discuss several things with patients, including [their] convictions. If you can’t do it, you’re in the wrong place. The suggestions about how I can start a discussion with patients about [their] convictions didn’t serve me in any way.’ (i1, n1)*

Participants stated that they greatly appreciated the expertise of the training provider and that the educator had done the proper background work and knew what she was talking about. They also noted that excellent teaching skills and the educator’s knowledge of complex cultural issues were meaningful. The ‘storytelling’ type of lecturing, and the high number of real-life examples that were presented in the sessions, were perceived as inspiring among the participants.

*‘It was so immersive, lively and multidimensional. Even though it was lecturing, it was somehow creative.’ (i3,n1)*

### Suggestions for training improvement

Participants brought up a few notable ideas that could make the training better in the future. Some noted that hearing about the lived experiences of persons from different immigrant groups could be added to the content. Some participants also suggested that the training could be slightly condensed. They felt pressure to finish their work on time to make it to the sessions, and many felt that four full afternoon sessions was too long to be outside the ward.

*‘It could have been a bit shorter, for instance by putting some material on the Web beforehand that could be used to orientate oneself and then having the face-to-face session where things would be summarised and discussed.’ (i2, n1)*

Participants also shared their opinions about the one-week break after each training session. Some participants felt that it allowed them to think about the contents of the sessions; but others felt that it was difficult to remember what had been previously discussed, which complicated the presentation of the big picture. Many participants stated that a shorter time span would have helped them to remember more clearly the content of a previous session and also helped them to assimilate the learned knowledge. They suggested that a summary from each session could have been provided.

The participants mostly felt that after the training, they no longer needed to use checklists or guidelines about how to act with certain patient groups. However, they still felt insecure about different religions and how the rules of different religions should be taken into account in their daily actions.

*‘We discussed how we encounter individuals, but not about how we respect different religious customs. For example, sometimes a male or female nurse is not allowed to help the patient with bathing, etc., or there are certain customs when it comes to end-of-life care.’ (i4, n1)*

## Discussion

In this study, we examined the healthcare professionals’ perceptions of the content, utility and implementation of cultural competence training that focused on easing cross-cultural encounters by increasing nurses’ awareness of their own culture and cultural biases. The prior expectations of participants regarding cultural competence training had to mainly do with acquiring certain ‘quick-fix’ solutions or guidelines on how to act with patients from different cultures. These thoughts matched with traditional cultural competence education, which focuses on providing knowledge about common ‘facts’ or the generalised behaviours of certain cultural groups [29]. However, this approach could have increased the risk of stereotyping and ignoring about the individual differences that patients with similar cultural backgrounds may have [30]. In the end, participants said they were extremely satisfied with the training, which provided them with a totally different perspective on the subject. Increasing awareness and gaining a better understanding of their own (Finnish) cultural and communicational features seemed to help them to recognise the common pitfalls of cross-cultural communication, and thus allowed them to develop their communication skills. This finding is in line with previous evidence suggesting that the first step towards improving cross-cultural communication is to raise awareness of one’s own verbal and nonverbal communication styles [11]. It is essential to realise that communicational differences can occur in how silences, pauses, eye contact, and touching are used and interpreted, or in how clear and direct messages are emphasised in different cultures (high- vs. low-context cultures) [31].

Interestingly, the participants in this study perceived it as an advantage that the training was not provided by their own healthcare organisation or by a healthcare professional. They stated that it was useful to have a different perspective on cultural issues, and they indicated that bringing new perspectives and ideas to the hospital environment from outside the healthcare field could facilitate the development of cross-cultural care. Continuing education is commonly provided by the hospital/organisation that employs healthcare professionals [32], and therefore utilising multiple perspectives by using professionals from different fields or organisations should be considered. Furthermore, the participants suggested that members of different immigrant groups could be invited to share their views in the training sessions. Participants believed they would thus achieve a better understanding of different cultures and how these patients experience the Finnish healthcare services. This so-called ‘educational partnership’ method, whereby different ethnic community members share their lived experiences, has previously been shown to provide an efficient way to increase healthcare professionals’ understanding of cultural differences and encourage further discussion [29]. Understanding the difficulties experienced by migrants could help professionals in increasing their cultural sensitivity and providing culturally competent care [33].

The importance of encouraging discussion about different cultural issues was highlighted in this study, and the participants commonly expressed a willingness to share their experiences and learned knowledge with their co-workers. The challenge was on how to pass on the valuable lessons learned to others in the organisation in such a way that the messages lying ‘behind the sentences’ could also be understood. Passing on information can be especially difficult in training settings that require one’s own critical thinking and a certain level of self-awareness of the theme in question. Participants noted that in order to develop current practices regarding cross-cultural care, the training should be provided to all healthcare professionals working at different organisational levels. The findings of this study are similar to previous findings, which state that organisational-level cultural competency initiatives, strategies and commitments are needed to provide culturally competent healthcare [5, 14].

Providing cost-effective training to a broader group of healthcare professionals would require utilising different educational methods, such as e-learning and technology-enhanced learning [34]. Despite the fact that the participants expressed appreciation for the face-to-face sessions with a storytelling-type of lecturing and discussions, they also had difficulties in detaching themselves from the busy wards and were stressed about being present and on time for all four training sessions. These difficulties, combined with irregular shift work, led to a decreasing number of participants in the sessions (approximately 12/20 participants were present per session). In addition, physicians were also invited to participate but none attended. This indicates that it can be difficult to arrange enough time in healthcare for this type of training and, therefore learning possibilities that are not bound to an exact time or place need to be further developed.

### Limitations

Certain issues place limitations on the credibility and transferability of the results. A single organisation and a small sample size (consisting mainly of nurses working in somatic wards) restrict the generalisation of the results. It is possible that other healthcare professionals (such as physicians, physiotherapists and mental health specialists) can have different perspectives on cultural awareness. Perceptions about the training could also have differed or be more multifaceted if all the nurses could have attended all four training sessions. Additionally, participants who enrolled in the training possibly were highly motivated to learn and had a more positive attitude towards cross-cultural care before attending the training, which might have affected their responses. It must also be considered that all the participants highlighted the teaching skills and experience of the educator; therefore their perceptions of the training could have been different if less competent educators would have been used. We did not ask for feedback from the participants about the data categorisation or interpretation of the results, which would have increased the trustworthiness of the results. However, two researchers were involved in the data collection and analysis, and frequent discussions were held with the research group during different phases of the study.

## Conclusion

There is clearly an international need to pay attention to the cultural competence of healthcare professionals. The results of this study indicate that increasing awareness of one’s own cultural features can be useful for easing cross-cultural encounters in a healthcare setting and improving the cultural competence of nurses. Participants expressed that the training was useful on many different levels, and they saw the small group size and inspiring lectures as important in facilitating discussion about cross-cultural care. In the future, it will be essential to provide cultural competence training to professionals at different levels of the healthcare system to increase their awareness of cultural differences and how culturally diverse patients are treated. Educational methods that would allow large groups to participate without restrictions on time and place are also needed. Future studies should compare traditional long-term training, such as the one used in the present study, to shorter training and Web-based learning platforms to find the most feasible way to increase cultural awareness and improve the cultural competence of healthcare professionals.

## Data Availability

Not applicable.
